# rDSA : an intelligent tool for data science assignments

**DOI:** 10.1007/s11042-022-14053-x

**Published:** 2022-11-05

**Authors:** Pierpaolo Vittorini, Alessandra Galassi

**Affiliations:** Department of Life, Health and Environmental Sciences, University of L’Aquila, P.le S. Tommasi, 1, Coppito, L’Aquila 67100 Italy

**Keywords:** Interactive learning environments, Automated feedback generation, Computer-assisted instruction, Automated assessment systems, Intelligent tutoring systems, Technology-enhanced learning

## Abstract

Tools supporting the teaching and learning of programming may help professors in correcting assignments and students in receiving immediate feedback, thus improving the solution before the final submission. This paper describes the rDSA tool, which was designed, developed, and evaluated to support students in completing assignments concerning (i) the execution of statistical analyses in the R language and (ii) commenting on the results in natural language. The paper focuses on the feedback provided by the tool to students and how it was designed/evaluated/improved over the years. The paper also presents the results of two studies that indicate the advantages of using the tool in terms of engagement and learning outcomes. To conclude, we provide a discussion on the characteristics of the tool, its use in similar courses, and the scope for future work.

## Introduction

Various tools that support professors and students in the teaching and learning of programming have been developed since the 1960s [15, 22]. Such tools may provide simplified development environments, use visualisations or animations to provide better insight into running a program, automatically grade student solutions, and guide students towards the correct solution through hints and feedback messages [24]. The manual correction of solutions is a complex, crucial, tedious, and error-prone task and the problem particularly aggravates when such an assessment involves many students. By automating the grading process, we assist teachers in making corrections, making it possible for students to receive immediate feedback, and improving on their solutions before submission.

In our previous research, we approached the problem of automated grading of assignments made up of a list of commands, their respective output, and comments (written in natural language) on the meaning of the output. We initially introduced a distance, expressed in terms of grade, between the correct solution (given by a professor) and the solution given by a student. Then, we focused on specific data science assignments, whose solutions required commands written in R language [10], the respective output and comments written in the Italian language. We then developed the rDSA (r Data Science Assignments) tool within the UnivAQ Test Suite (UTS) system^1^, which implemented the aforementioned distance for this kind of assignments [4, 7, 12, 18, 45].

More recently, we focused our attention on the feedback that the rDSA tool returns to students after automated grading. As known, feedback is a crucial element in learning [21, 30], and can be defined as “the process in which learners obtain information about their work [...], the qualities of the work itself, to generate improved work” [8]. Feedback is useful for both formative and summative assessments, i.e., in the execution of a course to verify the learning progress (by the teacher or by the students themselves), and at the end of the course to determine the learning outcomes [20]. Within a design-based research approach [6], the paper presents the three design/evaluation/improvement cycles about the feedback returned to students and reports on the possible improvements in the learning outcomes.

To present the research, we structured the paper as follows. Section 2 discusses the application scenario and foreseen educational impact. Section 3 summarises the related work. Section 4 describes the rDSA tool development and evaluation, in terms of three iterations and the respective results. Section 5 discusses the overarching findings of the paper and compares the results with previously published work. Section 6 concludes the paper by summarising the main results and presenting future work.

This study extends the results reported in [17] as follows. First, we analysed the state-of-the-art in more detail by providing more evidence on the effectiveness of the feedback during the learning process. Second, we presented all our contributions in terms of the three iterations that led to the current design and implementation of the rDSA tool. Third, we added a further evaluation cycle (i.e., online interviews), the novel implementation of the tool, and the learning outcomes.

## Application scenario and educational impact

Health Informatics as a course in Medicine and Surgery, as well as Information Systems in Nursing Sciences and Prevention Sciences in the University of L’Aquila (Italy), have a specific topic on how to execute statistical analyses in R and how to correctly interpret the results into the corresponding clinical findings. The related exercises and the final exam have the same structure: they start with the definition of the dataset and then list the analyses and technical/clinical interpretations that should be performed. The analyses must be executed through R commands and can be both descriptive (e.g., mean, sd), or inferential (e.g., t.test, wilcox.test), and test normality (e.g., shapiro.test). To interpret the results, students must understand, for example, if a test on normality suggests that the distribution should be normal or not, or if a test on a hypothesis is statistically significant.

**Fig. 1:**
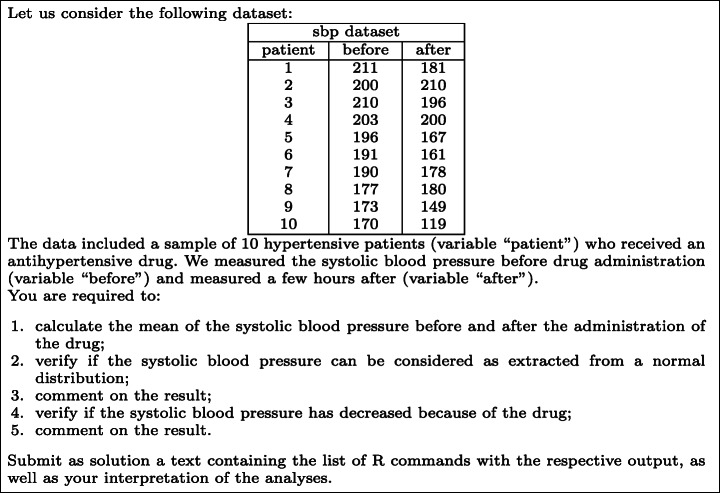
A sample exercise


For instance, see Fig. 1. The assignment regards a simple experiment about the effects of an antihypertensive drug. Let us consider the fourth point in the assignment. Since the systolic blood pressure was quantitative and the same patients were measured before and after the treatment, the student (after a normality test) should use a paired t-test. Such a test is executed in R using the command

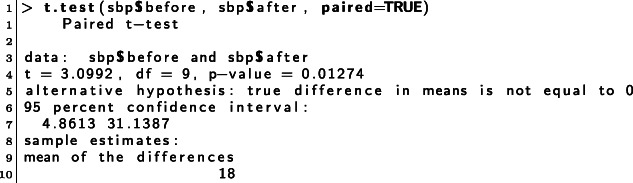


The p-value (see line 4 of the output) is 0.01274, less than 0.05. The student should then conclude that the difference in systolic blood pressure is statistically significant and therefore it should be caused by the effect of the drug. This conclusion solves point 5 of the assignment.

The experience gained during the years in correcting such assignments enabled the authors to identify the most common mistakes, which are of varying types and are sometimes difficult to spot. Given this educational scenario, we began the design and implementation of an automated grading tool that supports both formative and summative assessment activities as follows. As a formative assessment instrument, it can provide students with both textual feedback and an estimated evaluation of the quality of the submitted solution, and it enables teachers to monitor students’ progress with their homework. As a summative assessment instrument, the tool can be used by the teacher to shorten and improve manual correction activities. Accordingly, this may cause several expected educational benefits. For students, these include the ability to support their understanding of the commands and interpretation of the results. Students should be able to improve their final learning outcomes. Professors should expect fewer returning students. Moreover, in [45] we already showed the tool reduced professors’ workload in terms of both evaluation time and errors.

Appendix A summarises the most relevant considerations about the didactic organisation of Italian courses in general and the specific courses examined in this work (e.g., degree system, exam sessions), which are useful for readers unfamiliar with the Italian Academic system.

## Related work

We structured the related work as follows. First, we discussed the specific problem in the automatic grading of short-text answers (in our context, the comments given to interpret the commands). Then, we discussed the literature on automated assessment systems (AASs) and intelligent tutoring systems (ITSs), with specific regard to the system focusing on programming assignments (in our context, the analysis of the commands and output, as well as the returned feedback). Then, we summarised our research on the effectiveness of different releases of automated feedback. Lastly, we summarised previous work from the researchers in this field.

### Automated grading of short-text answers

Several solutions have been proposed to perform automated grading of short-text answers [9]. In such a context, the common task is to assign either a real-valued score (e.g., from “0” to “1”) or to assign a label (e.g., “correct” or “irrelevant”) to a student response. The approaches are manifold. Some rely on knowledge bases and syntactic analyses [31], which are available only for a limited set of languages. More recent studies have exploited the potential of vector-based similarity metrics to compare students’ answers against a gold standard [39, 45]. Such features were further explored in [29] to include several corpus-based, knowledge-based, word-based, and vector-based similarity indices. Recently, few studies have been conducted using transformer-based architectures [14] and neural network classifiers to perform short-answer grading [2, 28, 40]. Although neural approaches have demonstrated acceptable performance and generalisation capabilities across domains and languages, they typically require large amounts of data to learn an accurate classification model.

### AASs and ITSs

Nowadays, the available learning systems have different capabilities, including the specific characteristics traditionally assigned to AASs and ITSs. As known, AASs focus on assessing a student’s ultimate solution to an exercise or exam, with a grade or feedback report, to ease instructors from manually assessing many students. ITSs focus on helping students arrive at the solution by offering help at each step.

Automated assessment of student programming assignments was first attempted in the sixties [22], and an extensive set of tools has been developed to date [25, 38]. Worth summarising is a literature review by Keuning et al. [25], where the authors reviewed over 100 AASs and ITSs in the STEM field^2^ and categorised them in terms of (i) the nature of the feedback and (ii) which techniques are used to generate the feedback. In what follows, we summarise the most important results and introduce acronyms for all system characteristics to easily relate our tool and its features to them.

As for the nature of feedback, the reviewed tools offer either a simple or an elaborate one. Simple feedback (SF) is just a report on the achieved performance level (e.g., 75% of correct answers), or feedback that communicates whether a solution is correct or not, or that provides a description of a correct solution. By contrast, the elaborated feedback (EF), provides detailed support to students. It can provide hints on pre-requirements or how to approach the exercise, explanations on subject matters and examples illustrating concepts, knowledge about mistakes, and how to proceed. With specific regard to the mistakes, the available systems report to the student if the program does not produce the expected results (EF-ER), if it contains syntactic errors (EF-SY), if crashes (EF-CR), if they use different algorithms that are not accepted (EF-AL), if has style (EF-ST), or performance issues (EF-PE). In terms of supporting students on how to proceed, the summarised tools give hints on what the student should do to correct a mistake (EF-CM), describe the next step a student must take to come closer to a solution (EF-NS), and provide hints on how to improve an already correct solution (EF-IS).

Different techniques are also used to generate feedback. The general approaches are: (i) model tracing (TEC-MT); the tool analyses the process by which the student is solving a problem; (ii) constraint-based modelling (TEC-CBM); the tool only considers the current solution against pre-defined solution constraints (e.g. the solution provided by the professor, the presence of a for-loop); (iii) tutoring based on data analysis (TEC-TDA); the tool generates hints by using extensive sets of past student solutions. The domain-specific techniques, among the many, are: (i) static code analysis; the program is analysed without running it, to detect misunderstood concepts, the absence or presence of certain code structures, and to give hints on fixing these mistakes (TEC-SCA); and (ii) dynamic code analysis; running a program and comparing the output to the expected output (TEC-DCA).

Focusing on the context of computer science education, many tools support different language paradigms and provide elaborated feedback to enhance learning [36]. For example, recent AASs/ITSs support functional programming such as learning procedural programming [19] and object-oriented languages, that include Java [41] and C [33], as well as (web) scripting languages such as PHP, JavaScript, and Python [11, 26].

### Effectiveness of (automated) feedback

Feedback plays an important role in the learning process because it helps students identify their strengths and weaknesses and target the areas that need more work, encourage self-evaluation, and increase engagement [16, 34, 43]. Feedback from automatic assessment of students’ solutions is an important aid for students’ learning processes in self-study and distance learning, and has a positive effect on learning outcomes. [21, 30].

A recent meta-analysis of experiments on the effectiveness of human tutoring, computer tutoring, and no tutoring [44], reported the following: The author categorised feedback by computer tutoring systems in terms of its detail level, that is: (i) answer-based, a single and unique feedback about the wrong answer; (ii) step-based, a feedback provided for each step of the solution; and (iii) substep-based feedback, similar to the step-based case, but with a finer-grained level of details. Accordingly, the author tested the hypothesis that the effect size of human tutoring > substep-based > step-based > answer-based > no tutoring. The effect size measures the extent of association between two variables (in our case, the different tutoring method and its effectiveness): the larger the effect size, the stronger the association between the two variables. With respect to no tutoring, the main conclusions were: the effect size of human tutoring is *d* = 0.79 more effective and step-based tutoring is almost as good as human tutoring (*d* = 0.76). The substep-based tutoring, given that there are only a few statistically reliable studies – seems to have an effect size of 0.75 < *d* < 0.80, and answer-based tutoring is only *d* = 0.31 more effective.

### Previous work from the authors

In the last few years, we started the design, development, and testing of the UTS system, with the objective of providing a state-of-the-art system to support both formative and summative assessment in terms of: (i) classical testing [13] such as quizzes with multiple-choice questions, (ii) computerised adaptive testing [46] such as sequence of multiple-choice questions that “adapts” to the student, and (iii) automated grading for both code snippets and open-ended answers [4, 7, 12, 45].

In regard to automated grading, as explained in the previous section, we focused on assignments comprising commands, output, and short answers. Starting from the aforementioned literature, in [45], we introduced a general approach valid for assignments whose solutions are represented as a list of triples containing the command, its output, and a possible comment. In the proposed approach, the solution provided by a student is compared to the correct solution provided by the professor. Then, we defined the distance between the two solutions, which represents the final grade: the larger the distance, the lower the grade, and vice versa. The distance is based on the following possibilities: a student issued (i) a correct command that returned the correct output, (ii) a command that seems correct but returned an output different from that of the professor, (iii) missed the command, or (iv) correctly or incorrectly interpreted the result of the analysis. This approach was implemented in the rDSA tool, which focuses on the R language for the programming part and the Italian language for the open-ended answers. In particular, the tool uses static source code analysis for the R code snippets, a supervised neural classifier fed by sentence embedding and distance-based features for the open-ended answers, and assembles all results in structured feedback [2, 45]. It is worth remarking that the approach, besides its contextualisation of the R language and the health setting, can be applied to any course that includes data science assignments, whose solutions are made up of a sequence of commands, the output, interleaved with comments written in natural language that explain the results.

Nevertheless, the research reported in this paper focuses on the automated feedback returned to students, how it was cyclically designed, implemented and evaluated, and which were the possible improvements in the learning outcomes.

## rDSA tool: development and evaluation

**Fig. 2:**
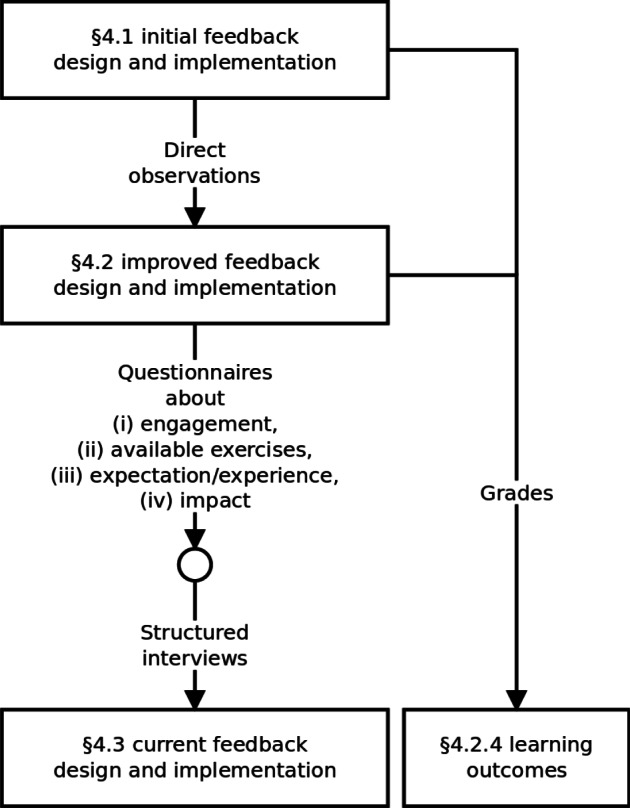
Structure of the iterative process

The objective of this section is to show the improvements of the rDSA tool, specifically, the automatic feedback, in terms of three iterations (see Fig. 2). The first iteration (Section 4.1) describes the initial feedback, its implementation, and its evaluation through direct observations. The feedback was then redesigned from the first implementation to provide complete, more structured, and detailed messages. The second iteration (Section 4.2) reports on the implementation of the improved design and on the three studies. The first two studies collected quantitative and qualitative data:

- through questionnaires, the first study measured: (i) the engagement, (ii) the usefulness, quality and relevance of the available exercises, (iii) the expectation/experience with the system, and (iv) the impact of the feedback in the learning process (Section 4.2.2);
- by means of structured interviews, the second study, collected useful suggestions – directly from students – on how to further improve the feedback (Section 4.2.3);

The third study focused on the learning outcomes of the students that used the initial and the improved feedback (Section 4.2.4). The third iteration (Section 4.3) shows the current feedback and the system architecture. The improved feedback was based on the suggestions and hints collected through the questionnaires and interviews completed by the students during the second iteration.

### First iteration

The first iteration is the initial step concerning the implementation and evaluation of the tool. The main results (Sections 4.1.1 and 4.1.2) were already published in [4, 18], and are briefly summarised here to help the readers understand all the improvements made to the rDSA tool.

#### First implementation

The first implementation of the rDSA tool [4] focuses on providing an automated evaluation of the solutions to the assignments, in terms of the final grade and basic feedback. The tool was mostly an AAS, returning both a simple feedback through final grade and an elaborate one with: (i) the correct commands, marked in green with a “Correct” statement; (ii) the commands that appeared correct but returned a wrong output, marked in blue with a “The command seems correct, but the output differs from the solution”; (iii) the missed commands, marked in red; and (iv) the student’s comments in green or red (if right or wrong, respectively), with a commenting statement. For the adopted techniques, we used (i) constraint-based modelling and static code analysis to analyse the commands and the related output, and (ii) the Levenshtein string similarity distance [27], divided by the length of the longest string^3^.

**Fig. 3:**
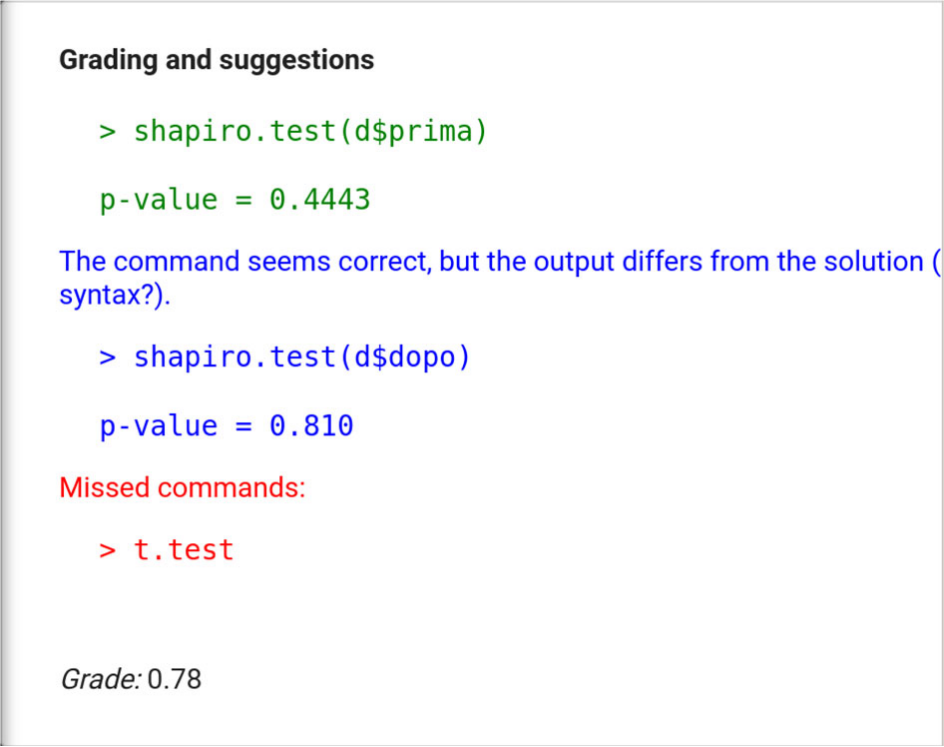
Student feedback – first implementation

As an example, let us consider the following solution to the exercise discussed in Fig. 1,^4^: 
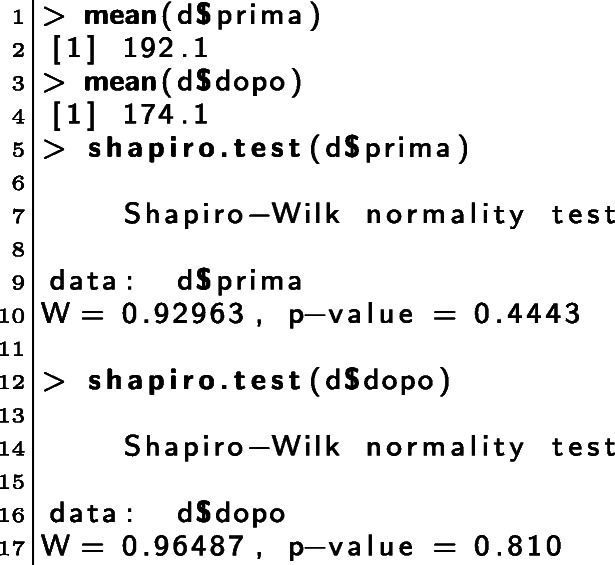


In the solution, the student omitted the commands to solve point 4 (because he/she did not issue the t.test command), executed correctly the mean s and only one of the shapiro.test s (the second one returned a wrong p-value), and did not give any interpretation of the tests. Fig. 3 shows the returned feedback, structured as described above, with the estimated grade ranging from 0 to 1.

#### First evaluation

By watching and listening to twelve students working with the tool, we collected all the highlighted issues and classified them into two categories [18]: (i) technical improvements and (ii) feedback structure. Focusing on the feedback, the students reported that the feedback was more technical than didactic because it only reported that the command was right or wrong, and failed to explain the error. Furthermore, the system automated grade leads to false pass/fail outcomes, especially when the grade is sufficiently close. To address these issues, we redesigned the feedback to cover all the possible cases of mistakes, as detailed in the following section.

#### Improved feedback design

The improved feedback was then designed as follows:

1. for each command given by the student
  - if the command and its output are equal to a certain command and output contained in the correct solution, return a “Correct” feedback;
  - if the command is in the correct solution, but its output differs from the output of the correct solution, we first return the generic message “The command seems correct, but the output differs from the right solution”, then we investigate the following two scenarios:
    1.b.1. the student made a mistake in the command call, by checking if the student used:
      - a wrong number of parameters: we either return the sentence “The parameter ... seems missing”, or the sentence “The parameter ... seems not needed”;
      - a wrong variable: we return the sentence “The variable ... does not seem correct”;
      - a wrong Boolean predicate for selecting a subset of rows: we return the sentence “The Boolean predicate ... does not seem correct”.
      Depending on the case, a message is added to suggest solutions for the error to the student. For instance, in the case of a t.test without the expected paired=TRUE parameter, we add the message “You should have used a paired test”;
    1.b.2. if nothing above applies, we assume that the student incorrectly imported the dataset and the message “Please check if the data was imported correctly” is returned.
  - the command is not in the correct solution. In this case, we first return the generic message “Wrong command” Then, we try to find in the correct solution a “similar” command, i.e., an inappropriate choice of command to calculate the central tendency or dispersion (referring to descriptive statistics) or the hypothesis testing (referring to inferential statistics). Depending on the case, a different message is returned. For example, if the student uses the median instead of the mean, we return the message “Another command to calculate the central tendency is in the correct solution. Did you misunderstand the question or variable type?”.
  - if the command requires a comment:
    1.d.1. if the comment is not present, we return the message “No comment was found”;
    1.d.2. if the comment is present:
      - if the comment is considered correct, we return the message “The interpretation of the analysis seems correct”;
      - else, we return the message “The interpretation of the analysis seems incorrect”.
2. all commands of the correct solution that were not identified in the previous analysis, are listed as “Missed commands”.

### Second iteration

#### Second implementation

The second implementation of the tool yields several improvements from both the technical and feedback viewpoints. We corrected the technical issues evident in the first evaluation and exploited novel methods for grading the comments as right or wrong, moving from the Levenshtein string similarity distance to supervised classification and sentence embedding [45]. For feedback, we implemented the previous design. In terms of the adopted techniques, together with constraint-based modelling and static code analysis for analysing the commands and the related output, we use tutoring based on data analysis to evaluate the comments.

As an example, let us consider the following solution to the exercise discussed in Fig. 1,^5^: 
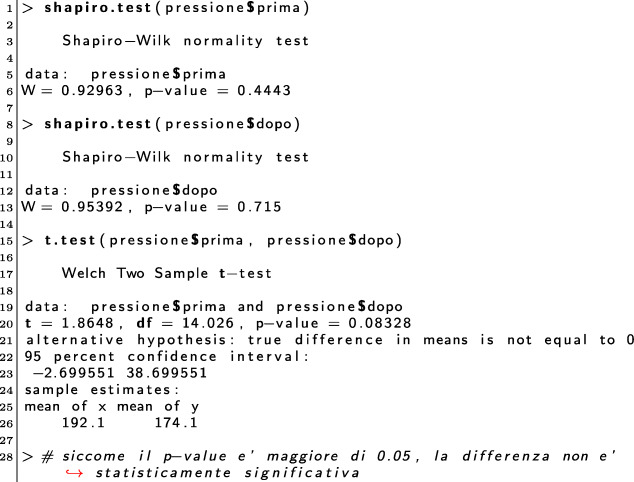


In the solution, the student omitted commands to solve point 1 (i.e., he/she did not issue the two required mean commands), executed correctly the shapiro.test, did not give an interpretation to the normality tests, forgot the paired=TRUE option for the hypothesis testing, but correctly interprets the (wrong) result.

**Fig. 4:**
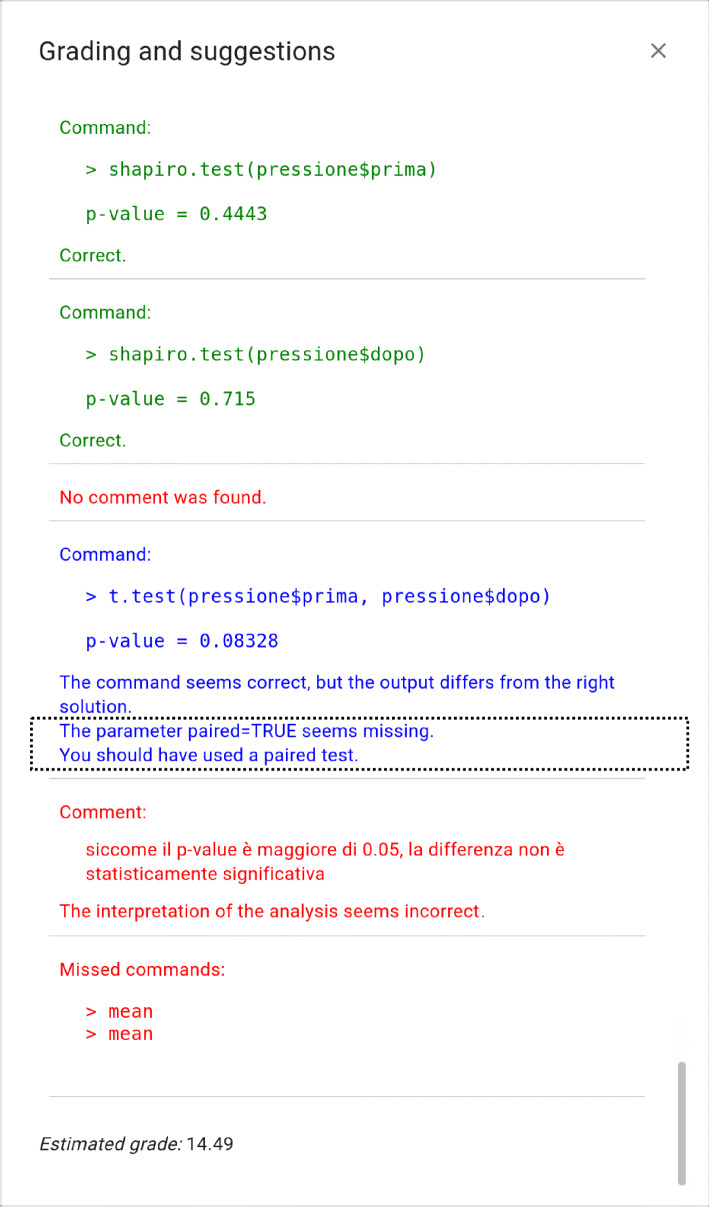
Student feedback – second implementation

Figure 4 shows the new feedback. The tool recognised the two correct normality tests (first two green blocks), but was unable to find their interpretation (the subsequent red block). It then found the t.test, but – given that the calculated p-value was different than that in the correct solution – the tool inspected the command call and found the missing paired=TRUE parameter. Hence, the tool reported such a problem in terms of three lines that close the blue block, the last two (highlighted in the figure) suggesting how to get to the correct solution. The tool automatically classified the comment given to the hypothesis testing as incorrect, as reported in the subsequent red block. In the last block, the tool reported the missing commands. The feedback is then completed with an estimation of the final grade, this time using the Italian grading system (i.e., from 0 to 30 cum laude).

#### Second evaluation (questionnaires)

##### Objective

The objective of the evaluation was to use standardised and ad-hoc questionnaires to verify the following: (i) the system improved the student engagement; (ii) the feedback was useful in helping students understand how to solve the exercises and deepen the grasp of the subject, how the students handled the system in preparation for the exam, that is, how they read through the exercises, or to check and submit the solution, or iteratively to refine the solution before the final submission; and (iii) if students would like to use similar tools in other subjects.

##### Materials and methods

**Fig. 5:**
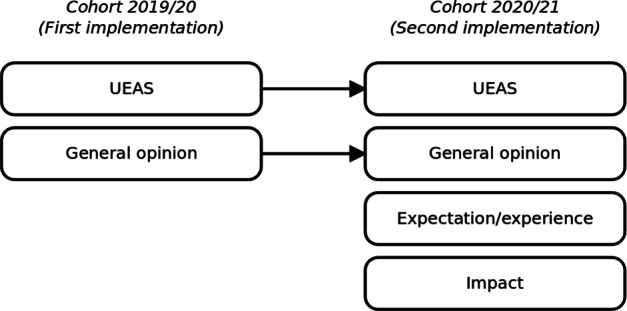
Study design

We conducted a study using data collected from two different cohorts, made up of students of Medicine and Surgery course, from the 2019/20 and 2020/21 academic years (see Fig. 5) and used the first and second implementation of the tool, respectively.

Both cohorts compiled the User Engagement Assessment Scale (UEAS [3]) and a question containing a general opinion on the feedback (see Appendix B). The 2020/21 cohort completed two further questionnaires. The first was structured to capture expectations and experiences on the following: (i) the usefulness of the feedback as a whole, (ii) the clarity of the explanations given for incorrect commands, (iii) clarity of the explanation given for the partially wrong commands, and (iv) usefulness in solving the exercise (see Appendix C). The second contained questions on the impact of the feedback, how feedback was implemented, and if they would recommend similar systems in similar subjects (see Appendix D).

The questionnaires were analysed:

- for UEAS, we scored the engagement as discussed in [3], then calculated the average engagement for both cohorts;
- as for expectation and experience questionnaire, we followed the approach proposed by Albert & Dixon [1]. We first calculated the mean of the expectations and experiences for each element. Then, we placed the results in a scatterplot (expectation on the x-axis, experience on the y-axis). As suggested in [1], elements in the top-right quadrant (i.e. good expectation and good experience) can be considered satisfactory; elements in the bottom-right quadrant such as good expectation and low experience need to be prioritised; elements in the top-left quadrants representing low expectation and good experience show a surprisingly good user experience; and elements in the bottom-left quadrant for low expectation and experience should be addressed as well, but with a lower priority.
- finally, all questions that required a Likert-scale answer were analysed through averages. For the multiple-choice questions, we used frequency tables.

Inferential analyses were performed using t-tests or Wilcoxon tests (depending on the type of variable – qualitative or quantitative – and if normally distributed or not), paired or not (in case of paired or independent samples) [35]. In the results, when reporting the p-value, we added an index that clarifies the adopted method, *w* for the Wilcoxon test, *t* for the t-test, and *p* for the paired version.

##### Results

40 students from the 2019/20 cohort and 16 students from the 2020/21 cohort answered the UEAS questionnaire. We observed an increased engagement, from 3.6/5 for the 2019/20 cohort, to 4.2/5 for the 2020/21 cohort, a statistically significant difference (*p*_*w*_ = 0.002).

The general opinion question investigated on usefulness, quality, and relevance of the available exercises. It was answered by 26 students in the 2019/20 cohort and 46 students in the 2020/21 cohort. In both cases, the general opinion was rated as 4.7/5.

**Fig. 6:**
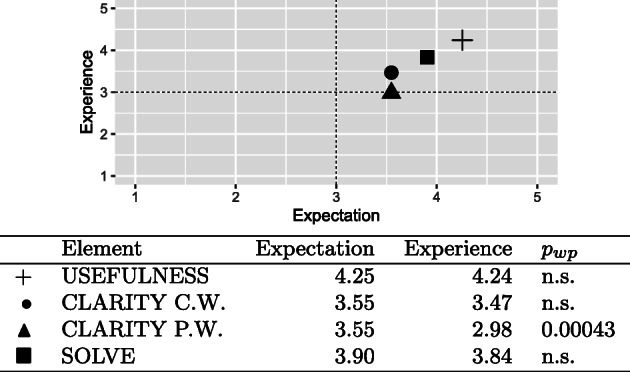
Result summary of the expectation/experience questionnaire administered to the 2020/21 cohort

A total of 63 students of the 2020/21 cohort answered the expectation/experience questionnaire. Figure 6 summarises the analysis of all questions, based on general usefulness (USEFULNESS), clarity of the feedback for the completely wrong commands (CLARITY C.W.), clarity for the partially wrong commands (CLARITY P.W.), and the usefulness for solving the exercise (SOLVE). The USEFULNESS, CLARITY C.W., and SOLVE elements are in the top-right quadrant and considered satisfactory. However, CLARITY P.W. – even if borderline – did not meet the expectations (*p*_*wp*_ = 0.00043).

Finally, the answers on the impact of the feedback showed that the system’s automatic feedback was useful for students to understand their errors (30 students), to understand the correct statistical method to solve the problem (37 students), and to verify the preparation for the final exam (36 students). Most of the students used the tool iteratively to improve their solutions (48 students). Few used the tool only before submitting the solution (12 students) or just to see the exercises (two students). Finally, students suggest using similar tools with a rate of 4.7/5.

According to the results, usefulness in general, clarity of the explanation for the completely wrong commands, and usefulness for solving the exercise had very positive expectations and experiences. On the other hand, the explanation for “the partially wrong commands” had an unsatisfactory experience. Improving this feedback element became a priority in our research: we therefore planned and conducted a further evaluation (Section 4.2.3), focusing on the explanatory feedback for partially wrong commands.

#### Second evaluation (interviews)

##### Objective

The objective of this evaluation was to collect suggestions from students on how to improve the explanatory feedback, especially for the partially wrong commands.

##### Materials and methods

To collect the suggestions from students, we used semi-structured interviews.

Interviews had different levels of structure. More structured interviews followed a strict and well-defined sequence of questions, whereas the unstructured interviews were more conversational, without following a fixed scheme. Structured interviews are usually easier to conduct and collect more consistent data than unstructured ones. On the other hand, unstructured interviews – at the expense of being more challenging and gathering less consistent data – may elicit suggestions or ideas coming from the interviewee that the interviewer may not have thought of.

We conducted our interviews online (due to the COVID-19 outbreak), as follows (see Appendix E):

- the interview was conducted with a conversational tone, one student at a time;
- before starting the interview, we asked for consent to record the interview and take pictures;
- during the interview we showed slides by sharing our computer screen;
- we structured our interview based on these seven elements; six identified cases of mistakes, and an “unknown” case;
- for each case, we first explained the case with an example (except for the “unknown” case), then we showed the feedback that the tool would have returned; hence, we asked:
  - a close-ended question asking if the returned feedback was clear or needed improvements;
  - an open-ended question asking how to improve the feedback.

The methods for the analysis of the open-ended questions were both narrative; making sense of the individual answers and thematic content analysis; identifying common themes between the different interviews.

##### Results

Six students volunteered for interviews. The results were as follows:

1. in case of a command given by a student, but is not explicitly required for the exercise and it is not a mistake, then the system should: (i) highlight it in orange, rather than in red (which is used for completely wrong commands), (ii) not consider it in the estimation of the final grade;
2. few students asked to directly receive the solution as feedback, rather than as suggestions on how to correct the error;
3. one student suggested structuring the feedback as a two-step process: in the first step, the feedback should include the suggestions as in the current version; in the second step – if the same error is repeated – the feedback should show the correct solution;
4. all students asked to improve the feedback to have more precise and punctual explanations.

Based on the suggestions reported above, we decided to improve the feedback as follows.

We accepted the first and the third suggestions, in terms of the two-step process. We therefore expanded the feedback design by adding a further step (that is, 1.b.3) before concluding the processing for partially wrong commands:

-
  - 1.b ...
    - 1.b.3 if a mistake is repeated for the same command, then return the correct command and the correct output.

Finally, for the fourth suggestion, we decided to take into account the propaedeuticity between commands (e.g., before issuing a parametric test, one should check the normality of the distributions) and if the used command is for a different type of study (e.g., a t-test used for more than two samples). For this suggestion, we expanded point 1.c of Section 4.1.3 as follows:

-
  - 1.c. the command is not in the correct solution. In this case, we first return the generic message “Wrong command” Then, we try to find in the correct solution a “similar” command, i.e., an improper choice of the command for calculating the central tendency or dispersion (referring to descriptive statistics) or the hypothesis testing (referring to inferential statistics). If a “similar” command is found, then
    - depending on the case, return an appropriate message. For example, if the student used the median instead of the mean, we return the message “Another command to calculate the central tendency is in the correct solution.”;
    - if propaedeuticities exist and are not respected, then, we return the message “Some preparatory commands seem to be missing”;
    - if the used command is appropriate for a different type of study, then, we return a message that explains the type of study the student is facing. For instance, if the student used a t.test instead of the aov (i.e. analysis of variance), we return the message “Please note that the study includes more than two samples.”;
    - else, we return the generic message “Did you misunderstand the question or the variable type?”

#### Learning outcomes

##### Objective

In this section, we report on the hypothetical effect of the tool in improving the didactic outcomes. We enrolled students only from the courses of Nursing Sciences and Prevention Sciences, because they both followed the course online (because of the COVID-19 pandemic), whereas the students from medicine and surgery followed the course either in presence (the 2019/20 academic year) or partially online (the 2020/21 academic year). In particular, we tested the following hypotheses:

- RQ.D.1 : Are the grades of students that attended the first session of exams of the 2020/21 academic year (on average) higher than the grades of the students that attended the first session of exams of the 2019/20 academic year?
- RQ.D.2 : Independently from the academic year, are the grades of students that used the tool for formative assessment 6 (on average) higher than the grades of students who did not use the tool?
- RQ.D.3 : Focusing only on the students that used the tool for formative assessment, are the grades of those that attended the first session of exams of the 2020/21 academic year (on average) higher than the grades of those that attended the first session of exams of the 2019/20 academic year?

##### Materials and methods

We collected the grades obtained by the students through the same set of eight different assignments; these were randomly assigned to each student by the UTS system during the aforementioned exam sessions. The analyses were both descriptive and inferential: means and standard deviations for descriptive statistics, t-tests, or Wilcoxon tests for inferential statistics. To choose between the t-test or the Wilcoxon test, we tested the normality of the distributions through a Shapiro-Wilk test [35]. Consequently, the p-value of the hypothesis testing is accompanied by either a *t* or *w* subscript to indicate that the reported value comes from a t-test or a Wilcoxon test, respectively.

##### Results

The results concerning RQ.D.1 are summarised in Table 1(a). The 2019/20 cohort comprises 67 students, whereas the 2020/21 cohort comprises 45 students. The average grade for the 2019/20 cohort is 23.88, the average grade for 2020/21 is 26.38. The difference was statistically significant (p value _*w*_ = 0.012). Regarding RQ.D.2 (see Table 1(b)), only 35 students did not use the tool, 77 did. On average, the grades increased from 22.65 to 25.90.

**Table 1:** Results about the didactic outcomes

Academic year	N	Mean(SD)	*p-value*_*w*_
(a) RQ.D.1			
2019/20	67	23.88(6.11)	0.012
2020/21	45	26.38(4.99)	
(b) RQ.D.2			
*Tool use*	*N*	*Mean(SD)*	*p-value*_*w*_
No	35	22.65(6.24)	0.002
Yes	77	25.90(5.32)	
(c) RQ.D.3			
	*N*	*Mean(SD)*	*p-value*_*w*_
2019/20	51	24.96(5.52)	0.009
2020/21	26	27.74(4.45)	

### Third iteration

#### Current implementation

**Fig. 7:**
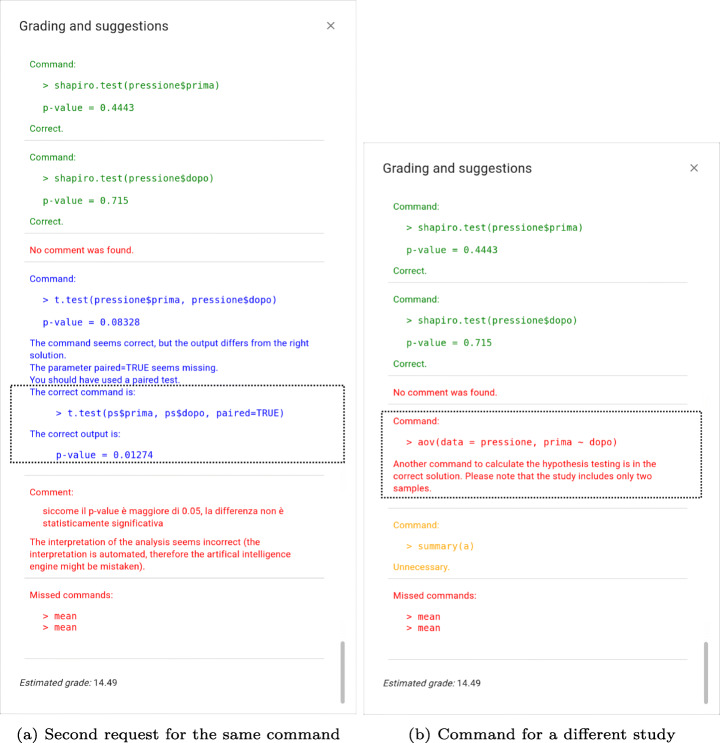
Student feedback – current implementation

Starting from both suggestions and opinions collected from the interviews Section 4.2.3 and questionnaires Section 4.2.2, the new feedback was implemented as follows. Figure 7(a) shows Case 1.b.3, discussed in Section 4.2.3. In particular, the dotted box highlights the part resulting from two subsequent calls of feedback (compared with Fig. 4, which does not contain this further explanation). Finally, Fig. 7(b) depicts one of the cases discussed in 1.c, and in particular, the case in which a “similar” command is issued that, however, should be used for a different study design. In the specific example, the student used the aov command instead of the t.test command.

#### Current architecture

**Fig. 8:**
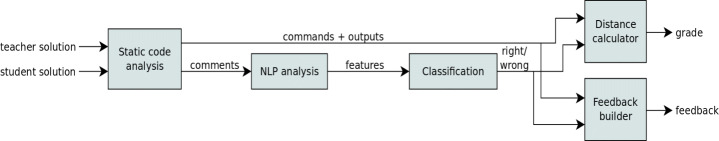
Tool architecture and feedback implementation

Figure 8 depicts the rDSA tool architecture. A solution is analysed as follows. First, the code is parsed, and the commands, outputs, and comments are extracted. Then, the comments are first processed by a Natural Language Processing (NLP) module to extract all relevant features, then classified as either right or wrong through a supervised classifier (see [2, 45] for details). Hence, the distance between the student’s and the teacher’s solution is calculated by the “Distance calculator” module. The distance is then converted to the estimated grade. Unlike the previous implementations, now the tool has a specific module (called “Feedback builder”) that takes care of assembling the feedback according to the aforementioned design. Technically, the system is developed in Java, uses Java-Server Faces [37] for implementing the user interfaces, Java Persistence API [23] for storing the data into a MariaDB database [5], Python scripts for the NLP analysis [42], and R as backend for the classification task [10].

## Discussion

The tool evolved during the three iterations as summarised in Table 2, with respect to the characteristics introduced in Section 3.2. The first release of the rDSA tool provided both the final grading (SF) and a short report about the wrong/correct commands/outputs/comments (EF-ER, GR-AB) by comparing the student’s solution with the correct one (TEC-CBM). From a technical viewpoint, rDSA uses static code analysis to evaluate the code (TEC-SCA). In the second iteration, the rDSA tool provided elaborated feedback to correct a mistake (EF-CM) and the next step towards the correct solution (EF-NS) by means of hints for each command containing a mistake (GR-SB). The second implementation also involved an automated classification of the comments as right/wrong, based on natural language processing and machine learning techniques (TEC-TDA). Finally, the last iteration added a more detailed feedback (EF-AL, GR-SSB).

**Table 2:** Summary of the characteristics of the rDSA tool

Iteration	SF	EF									TEC					GR
		ER	CM	NS	AL	SY	CR	ST	PE	IS	CBM	SCA	TDA	DCA	MT	
1st	×	×									×	×				AB
2nd	×	×	×	×							×	×	×			SB
3rd	×	×	×	×	×						×	×	×			SSB

SF : Simple feedback EF : Elaborated feedback EF-ER : Elaborated feedback - expected results EF-CM : Elaborated feedback - how to correct a mistake EF-NS : Elaborated feedback - next step EF-AL : Elaborated feedback - different algorithm EF-SY : Elaborated feedback - syntactic errors EF-CR : Elaborated feedback - crashes EF-ST : Elaborated feedback - style EF-PE : Elaborated feedback - performance issues EF-IS : Elaborated feedback - improve a correct solution TEC-CBM : Techniques - constraint-based modelling TEC-SCA : Techniques - static code analysis TEC-TDA : Techniques - tutoring based on data analysis TEC-DCA : Techniques - dynamic code analysis TEC-MT : Techniques - model tracing GR : Granularity GR-AB : Answer-based GR-SB : Step-based GR-SSB : Substep-based

The analyses presented in the previous section yield several interesting results. The first regards the increased engagement by the 2020/21 cohort with vis-a-vis the previous cohort. This result supports our hypothesis, that more detailed and explanatory feedback could raise more attention and participation in students. Nevertheless, the 2019/20 cohort followed the lectures in class, whereas the 2020/21 was online, creating a clear bias. However, at this point, we do not have two cohorts that can be compared without biases. We therefore considered this result preliminary, as it required verification, nevertheless encouraging.

Furthermore, the fact that a majority of the students used the tool iteratively as a guide (confirming or suggesting changes), refining the solution until the final submission, suggesting using similar tools, showed the key role played by the tool in exam preparation.

The analysis of the expectation/experience was instrumental to highlight the successful three elements of the automated feedback (i.e., usefulness in general, clarity of the explanation for the completely wrong commands, and usefulness for solving the exercise) and the unsatisfactory one (i.e., the explanation for “the partially wrong commands”). It was also helpful in defining the priority and suggesting a further evaluation focusing on the explanatory feedback for partially wrong commands. It is worth noting, these types of errors are the most deceptive and ambiguous. The student knew which command had to be used, but introduced “something” wrong in the call (e.g., a wrong variable or wrong data). Therefore, an explanation that solves these types of errors must be precise and specific to be effective. In other words, explaining a completely wrong command is somewhat easy: for example, the use of a median instead of a mean can easily be explained in terms of an incorrect choice of the central tendency indicator, while a command that appears correct but returns a different output may be caused by a multitude of factors, sometimes difficult even for a teacher to spot. Therefore, the initial identification of the possible cause, the generation of automated feedback that explains that specific mistake and suggests a way to solve the issue is actually a difficult task to implement.

The results of the didactic outcomes are very positive. All comparisons were statistically significant, suggesting a positive effect of the tool and automated feedback. However, there are at least two limitations. First, the 2019/20 and 2020/21 cohorts included different students that followed the course in the classroom or online (due to COVID-19 pandemic), respectively. Nevertheless, the students in the different years came from the same type of courses, and were admitted to the university after passing the same type of admission test, following the same teaching program in the same modality. Second, the students who decided to use the tool might be more motivated and thus, in advance, more likely to get higher grades than the others.

## Conclusions

This paper summarises our latest research on the design, development, and evaluation of the feedback provided by the rDSA tool. In previous research, we focused on the more technical aspects of the tool, i.e., code analysis, automated grading of short-text answers, and their implementation. Only recently, we started focusing on the feedback and how to structure it, to be effective in improving the comprehension of the subject and – as a consequence – the solutions given to assignments.

To this aim, we conducted several studies that yielded interesting results. Increased engagement and learning outcomes were observed in students who used the second release instead of the first release. In addition to the previously discussed limitations, the results are in line with the literature summarised in Section 3.3 and suggest the effectiveness of adopting the rDSA tool as a formative assessment instrument. Continuing this research line, we are planning a further evaluation step concerning the latest implementation and its effectiveness in explaining the most ambiguous and insidious mistakes.

Several features can still be added with respect to the state-of-the-art. The most pertinent ones are the analysis of syntax errors (EF-SY) and the possibility of offering personalised hints based on a student model (TEC-MT) [32]. Less relevant are the performance analysis (EF-PE) and how to improve an already correct solution (EF-IS). So far, performance is not a key factor in the assignments we are currently providing to students. If we introduce topics requiring it in our lectures (e.g. deep learning, dealing with large datasets), this characteristic will become a priority. A similar consideration stands for how to improve an already correct solution. Thus far, given our assignments and the practical aim of the course, a correct solution can be potentially improved only by using a different command. Not pertinent to our type of assignments are the remaining ones, that is, dealing with crashes (EF-CR), style (EF-ST), and using dynamic code analysis (TEC-DCA).

However, it is worth mentioning the effort devoted to the implementation. Developing the static code analysis module, collecting the gold standards and short answers given by students, setting up the natural language module as well as the supervised classifier, requires significant effort. Therefore, a researcher/professor wishing to introduce a similar tool within his/her teaching activities must ponder and reflect on these points.

To help researchers, the rDSA source code and datasets used in this research were made publicly available on the following website: https://vittorini.univaq.it/uts/

## Appendix A: Didactic organisation

Teaching is organised as follows:

- the Academic year begins in Autumn and lasts 12 months;
- the first day is October 1^*s**t*^ and the last day is September 30^*t**h*^ of the successive year;
- the Academic year is divided into two semesters;
- usually, the first semester starts in October and ends in February, the second starts in March and ends in July;
- a course can be held in a semester or during the entire year.

Exams are organised as follows:

- there are multiple exam sessions during the Academic year;
- usually, there are 3 sessions per year: one at the end of the first semester (February), another at the end of the second semester (July), and the last one before the beginning of the successive Academic year (September);
- a student can take the same exam multiple times until he/she passes it;
- exam grades range from 0 to 30 cum laude, customarily considered as 31;
- an exam is passed with a grade ≥ 18.

Referring to the specific courses of “Health Informatics” in Medicine and Surgery, and “Information Systems” in Nursing Sciences and Prevention Sciences:

- both courses are numerus clausus. The following table summarises the size of each cohort per degree course:
  **Table:**
  Degree course	2019/20	2020/21
  Medicine and Surgery	137	137
  Nursing Sciences	49	49
  Preventive Sciences	30	30
  *Total*	216	216
- the course of Health Informatics is in the first semester, the course of Information System is in the second semester;
- the use of the rDSA tool is recommended for formative assessment;
- the rDSA tool is used for summative assessment, without the automated feedback;
- the exercises solved as homework are not considered for the final grade.

## Appendix B: General opinion

**Table:**

	1	2	3	4	5
*1=Useless, 2=Not very useful, 3=Neutral,*					
*4=Useful, 5=Extremely useful*					
How do you evaluate the usefulness, quality, and relevance of the exercises available on the formative assessment tool for exam preparation?					

## Appendix C: Expectation/experience questionnaire

**Table:**

	1	2	3	4	5
*1=Useless, 2=Not very useful, 3=Neutral,*					
*4=Useful, 5=Extremely useful*					
How did you expect the automatic feedback provided by the platform to be in general?					
How do you generally rate the automatic feedback provided by the platform?					
*1=Not at all, 2=A little, 3=Neutral, 4=Very, 5=Completely*					
For completely wrong commands, that is, those highlighted in red, how much did you expect the feedback provided by the platform to be clear?					
For completely wrong commands, that is, those highlighted in red, how clear did you find the feedback provided by the platform?					
For partially wrong commands, that is, those highlighted in blue, how much did you expect the feedback provided by the platform to be clear?					
For the partially wrong commands, that is, those highlighted in blue, how clear did you find the feedback provided by the platform?					
*1=Surely not, 2=No, 3=Maybe, 4=Surely yes, 5=Yes*					
Did you expect that the explanation of the feedback would allow you to solve the exercise correctly?					
Did the explanation of the feedback later allow you to solve the exercise correctly?					

## Appendix D: Impact

**Table:**

*Check all those that apply*					
The automated feedback provided by the platform allowed me to:					
1. Understand my mistakes					
2. Understand the correct statistical method for solving the problem					
3. Verification of my preparation for the final exam					
*Select only one*					
How did you use the system?					
1. I only used it to see the exercises					
2. I only use it before submitting the solution					
3. I used it iteratively, to improve my solution before the final submission					
	1	2	3	4	5
*1=Surely not, 2=No, 3=Maybe, 4=Surely yes, 5=Yes*					
Would you recommend the use of automatic feedback systems of this type to prepare for similar examinations?					

## Appendix E: Interview

**Table:**

A) Wrong variable
*Example.* Suppose we ask you to calculate the average weight of a few patients and run the following command:
> mean(es$height)
*Feedback.* The system would reply to you: “The command looks correct, but the output is different from the right solution; the variable height does not seem correct”
*Questions.* (a) According to your opinion, can feedback improve? (b) In the affirmative case, how do you suggest improving it?
B) Wrong boolean predicate
*Example.* Suppose we ask you to calculate the weight of female patients and run the command:
> mean(es$weight[es$sex=="M"])
*Feedback.* The system would reply to you: “The command looks correct, but the output is different from the right solution; the Boolean predicate sex=="M" does not seem correct”
*Questions.* (a) According to your opinion, can feedback improve? (b) In the affirmative case, how do you suggest improving it?
C) Missing parameter - pt 1
*Example.* We asked you to calculate if there was a statistically significant drop in cholesterol after taking a new statin. You run the command:
> t.test(es$col.pre, es$col.post)
*Feedback.* The system would reply: “The command looks correct, but the output is different from the right solution; the parameter paired=TRUE appears to be missing; you should have used a paired data test”
*Questions.* (a) According to your opinion, can feedback improve? (b) In the affirmative case, how do you suggest improving it?
D) Missing parameter - pt 2
*Example.* Suppose we ask you to calculate if there is a statistically significant difference in cholesterol between men and women. You run the command:
> t.test(es$col, es$hdl)
*Feedback.* The system would reply: “The command looks correct, but the output is different from the right solution, the parameter col[sex=="M"] appears to be missing; the parameter col[sex=="F"] appears to be missing; the hdl variable does not seem correct”
*Questions.* (a) According to your opinion, can feedback improve? (b) In the affirmative case, how do you suggest improving it?
E) Too many parameters
*Example.* Suppose we ask you to calculate if there is a statistically significant difference in cholesterol between men and women. You run the command:
> t.test(es$col[es$sex=="M"], es$col[es$sex=="F"], paired=TRUE)
*Feedback.* The system would reply: “The command looks correct, but the output is different from the right solution, the parameter paired=TRUE does not seem necessary; you shouldn’t have used a paired data test”
*Questions.* (a) According to your opinion, can feedback improve? (b) In the affirmative case, how do you suggest improving it?
F) Similar command
*Example.* Suppose we ask you to calculate the average weight of some patients. You run the command:
> median(es$height)
*Feedback.* The system would reply to you: “The command looks correct, but the output is different from the right solution. In the correct solution, a different command was used to calculate the centre tendency. Please check the question and type of variable. ”
*Further note for the student.* Similar feedback is returned in the case of confidence intervals (e.g. ci.mu.t instead of ci.median) and for the hypothesis test (e.g. t.test instead of wilcox.test)
*Questions.* (a) According to your opinion, can feedback improve? (b) In the affirmative case, how do you suggest improving it?
G) Unknown case
*Feedback.* When the system cannot find a better explanation for a command that looks correct but gives a wrong result, the system responds as follow: “The command looks correct, but the output is different from the right solution. Please check if the data have been imported correctly”
*Questions.* (a) According to your opinion, can feedback improve? (b) In the affirmative case, how do you suggest improving it?

## Footnotes

1 Available at URL https://vittorini.univaq.it/uts/.

2 STEM is a well-known acronym for the fields of science, technology, engineering, and mathematics. It is commonly used as a general container for all scientific and technological subjects.

3 This division is adopted for the comments to return a distance in the range [0,1].

4 English for Italian sentences:
(i) *“Pressione sistolica”* : Systolic blood pressure (ii) *“prima”* : before; (iii) *“dopo”* : after.

5 English for Italian words/sentences: (i) *“pressione”* : blood pressure; (ii) *“prima”* : before; (iii) *“dopo”* : after; (iv) *“siccome il p-value e’ maggiore di 0.05, la differenza non e’ statisticamente significativa’* : given that the p-value is larger than 0.05, the difference is not statistically significant.

6 We considered that a student used the tool or not if he/she completed at least half of the available exercises.
